# Supplementary material for: In-Situ Synthesis of Methyl Cellulose Film Decorated with Silver Nanoparticles as a Flexible Surface-Enhanced Raman Substrate for the Rapid Detection of Pesticide Residues in Fruits and Vegetables
Source: Materials (Basel). 2021 Oct 1;14(19):5750. doi: 10.3390/ma14195750 (PMC8510044; doi:10.3390/ma14195750)
Supplement: Supplementary file 1 [file materials-14-05750-s001.zip › materials-1379095-supplementary.pdf]

Supplementary Information

In-situ synthesis of methyl cellulose film decorated with silver nanoparticles as a flexible surface-enhanced Raman substrate for rapid detection of pesticide residues in fruits and vegetables

Qijia Zhang [1], Guangda Xu [1], Na Guo [1], Tongtong Wang [1], Peng Song [2,*] and Lixin Xia [1,*]

1 College of Chemistry, Liaoning University, Shenyang 110036, China; zqj13940305120@163.com (Q.Z.); xuguangdalnu@163.com (G.X.); 18404713138@163.com (N.G.); 18340312652@163.com (T.W.)
2 Yingkou Institute of Technology, Yingkou 115014, China
* Correspondence: songpeng@lnu.edu.cn (P.S.); lixinxia@lnu.edu.cn (L.X.); Tel.: +86-24-62202258 (L.X.)

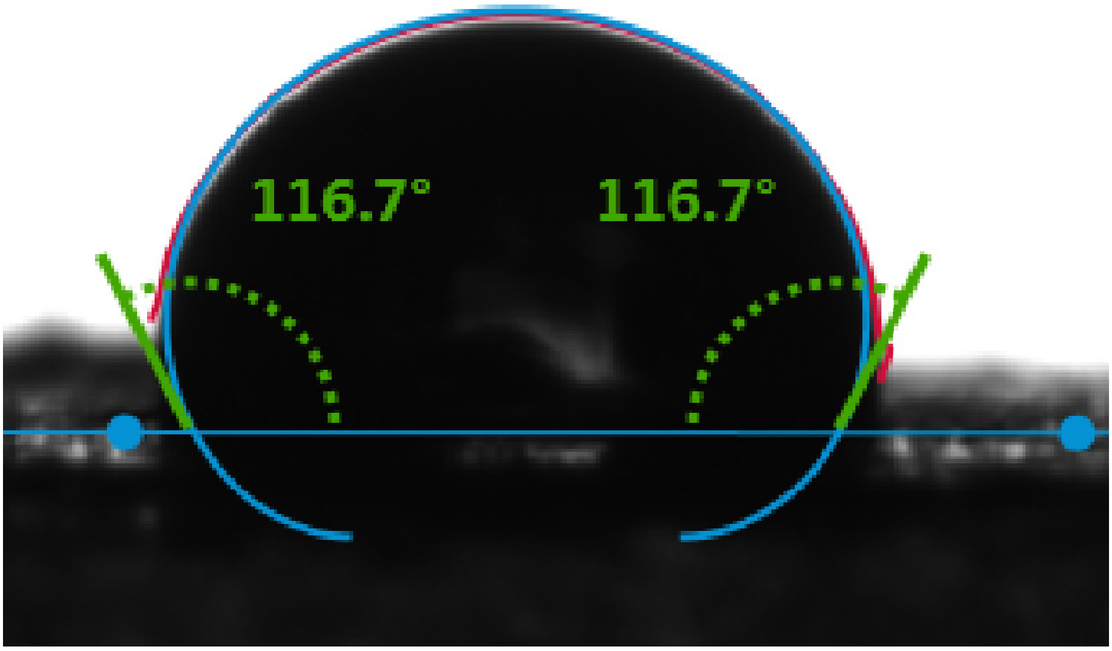

Figure S1. Contact Angle of MC/Ag NPs film.

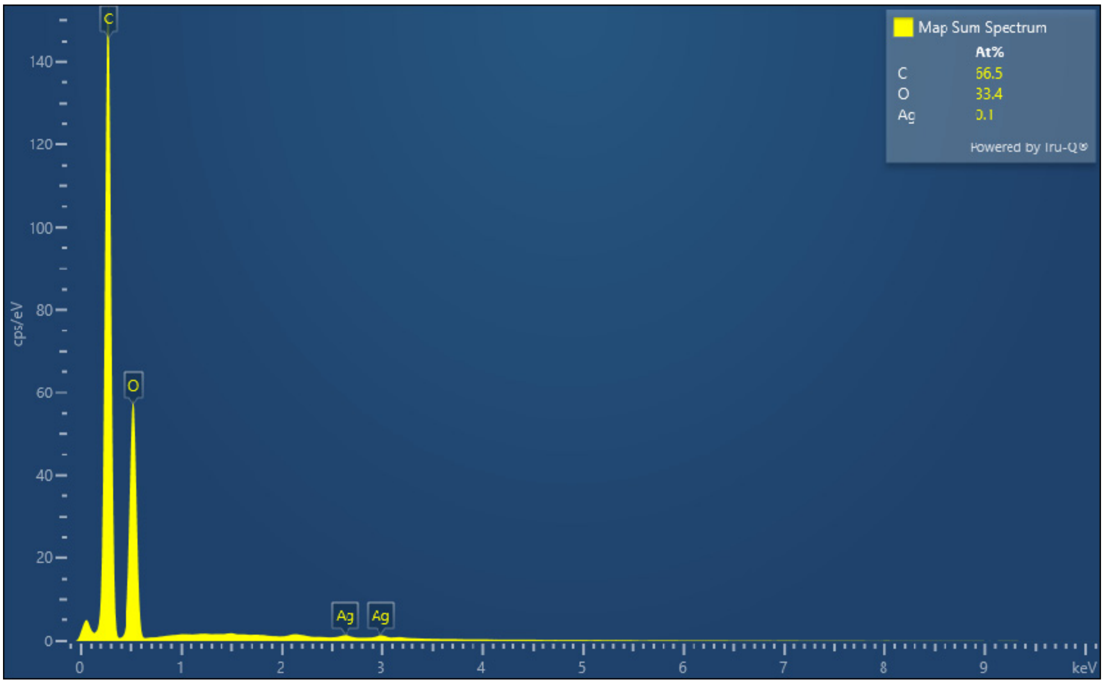

Figure S2. EDS image of MC/Ag NPs film SERS sute.bstra.
